# Intravenous vitamin C administration to patients with septic shock: a pilot randomised controlled trial

**DOI:** 10.1186/s13054-022-03900-w

**Published:** 2022-01-25

**Authors:** Patrice Rosengrave, Emma Spencer, Jonathan Williman, Jan Mehrtens, Stacey Morgan, Tara Doyle, Kymbalee Van Der Heyden, Anna Morris, Geoff Shaw, Anitra C. Carr

**Affiliations:** 1 Department of Pathology and Biomedical Science, University of Otago, Christchurch, PO Box 4345, Christchurch, 8140 New Zealand; 2 Centre for Postgraduate Nursing Studies, University of Otago, Christchurch, PO Box 4345, Christchurch, 8140 New Zealand; 3 Department of Population Health, University of Otago, Christchurch, PO Box 4345, Christchurch, 8140 New Zealand; 4 Department of Intensive Care Medicine, Christchurch Hospital, Private Bag 4710, Christchurch, 8140 New Zealand

**Keywords:** Septic shock, Sepsis, Vitamin C, Noradrenaline, Vasopressor, ICU length of stay

## Abstract

**Background:**

Intravenous vitamin C administration in septic shock may have a sparing effect on vasopressor requirements, and vitamin C’s enzyme cofactor functions provide a mechanistic rationale. Our study aimed to determine the effect of intravenous vitamin C administration on vasopressor requirements and other outcomes in patients with septic shock.

**Methods:**

This was a double-blind, randomised placebo-controlled trial in 40 patients with septic shock who were randomised (1:1) to receive intravenous vitamin C (at a dose of 25 mg/kg of body weight every 6 h) or placebo (intravenous 5% dextrose) for up to 96 h, or until death or discharge. The primary outcome was intravenous vasopressor requirements (dose and duration), and secondary outcomes included Sequential Organ Failure Assessment (SOFA) scores, intensive care unit (ICU) and hospital length of stay, and mortality. In addition, blood samples were collected to determine vitamin C kinetics and inflammatory marker concentrations.

**Results:**

Median plasma vitamin C concentrations were deficient at baseline (9.2 [4.4, 12] µmol/L) and increased to 408 (227, 560) µmol/L following 72 h of intervention. The mean duration of intravenous vasopressor infusion in the vitamin C group was 48 (95% CI 35–62) hours and in the placebo group was 54 (95% CI 41–62) hours (p = 0.52). The dose of vasopressor delivered over time was comparable between the two groups, as were SOFA scores (p > 0.05). The median ICU length of stay in the intervention group was 3.8 (2.2, 9.8) days versus 7.1 (3.1, 20) days in the placebo group (p = 0.12). The median hospital length of stay for the vitamin C group was 18 (11, 35) days versus 22 (10, 52) days for the placebo group (p = 0.65). Mortality was comparable between the two groups (p > 0.05). Of the inflammatory markers, neutrophil counts were elevated in the vitamin C group relative to placebo by 72 h (p = 0.01). C-reactive protein and myeloperoxidase concentrations were elevated at baseline, however, the two groups were comparable over time (p > 0.05).

**Conclusions:**

Our pilot study indicated that intravenous vitamin C did not provide significant decreases in the mean dose or duration of vasopressor infusion. Further research that takes into account the potential impact of intervention timing, dose and duration, and location of trial, may provide more definitive evidence.

***Trial registration*:**

ACTRN12617001184369 (11/8/2017).

**Supplementary Information:**

The online version contains supplementary material available at 10.1186/s13054-022-03900-w.

## Background

Sepsis is a life-threatening condition comprising organ dysfunction due to a dysregulated host response to infection [1]. Sepsis is a growing global health issue, with an estimated 49 million cases worldwide resulting in 11 million deaths [2]. As such, sepsis is responsible for 20% of annual global deaths. Septic shock is a more severe clinical presentation characterised by profound circulatory, cellular, and metabolic abnormalities and is associated with mortality rates greater than 40% [1]. Sepsis is managed through empiric intravenous antibiotic therapy, source control of infection, fluid resuscitation, and vasopressor administration. Additionally, organ support is provided via mechanical ventilation and renal replacement therapy [3]. Intravenous norepinephrine is usually the first-choice vasopressor to increase the mean arterial blood pressure ≥ 65 mmHg for patients with sepsis who remain hypotensive after adequate fluid resuscitation. At times, intravenous vasopressin is also commenced to aid mean arterial pressure target or decrease norepinephrine dosage.

Since the publication of several early trials indicating possible beneficial effects of intravenous vitamin C in critically ill patients with sepsis and septic shock, as monotherapy [4, 5] and in combination with thiamine and hydrocortisone [6], there has been an upsurge in clinical trials investigating its potential benefits in these patients [7]. Various trials administering intravenous vitamin C to patients with sepsis and septic shock have indicated decreased vasopressor requirements [5, 8–10], improved sequential organ failure assessment (SOFA) scores [4, 9], decreased ICU length of stay [8, 11], and decreased mortality [5, 9, 11]. However, some trials showed no effects of vitamin C intervention on SOFA scores [11], ICU length of stay [5], or mortality [8, 10], so the evidence is currently mixed with regard to these outcomes. Since the emergence of severe coronavirus disease in 2019 (COVID-19), research into the potential treatments of sepsis, a major complication of severe COVID-19, has become even more important. Early trials have indicated some potentially beneficial effects of intravenous vitamin C in severe COVID-19 [12–14]. In addition, the World Health Organisation has highlighted vitamin C as a potential adjunctive therapy with biologic plausibility for patients with critical COVID-19 [15].

Vitamin C has pleiotropic mechanisms of action that could plausibly contribute to its beneficial effects in sepsis and severe COVID-19, such as antioxidant, anti-inflammatory, antithrombotic, and immuno-modulatory functions, including roles in leukocyte and platelet functions, and endothelial and epithelial cell integrity [16, 17]. Patients with sepsis and septic shock have low vitamin C status and a high prevalence of deficiency [18, 19], and these critically ill patients require gram doses of intravenous vitamin C to replete their plasma status [20, 21]. In 2015 we proposed that vitamin C’s role as a cofactor for the endogenous synthesis of vasopressors (norepinephrine and vasopressin) may provide a rationale for its administration in septic shock as these vasopressors are routinely administered to critically ill patients to try and increase their blood pressure [22]. Shortly afterwards, Zabet et al. [5] published a paper supporting this premise, reporting a decrease in both dose and duration of noradrenaline administration in patients with septic shock who received intravenous vitamin C at a dose of 100 mg/kg body weight per day. We initiated a pilot double-blind, randomised-controlled trial to ascertain if these findings were reproducible.

## Methods

### Patient enrolment

This was a double-blind, randomised placebo-controlled trial of intravenous vitamin C infusion in patients with septic shock at Christchurch Hospital ICU, Christchurch, New Zealand. Ethical approval for the study was obtained from the New Zealand Northern A Health and Disability Ethics Committee (16NTA238). The study was registered with the Australia and New Zealand Clinical Trial Registry (ACTRN12617001184369). Proxy consent was obtained from the treating physician in consultation with the next of kin when patient consent was not immediately possible. Written informed consent from the patients was sought as soon as they had sufficiently recovered.

Patients with septic shock were screened on admission to the ICU. They were enrolled into the study if they met the following criteria: receiving intravenous antimicrobial therapy specifically for infection, receiving ≥ 5 µg/minute (≥ 0.06 µg/kg/min) noradrenaline or adrenaline, evidence of organ dysfunction, i.e. Sequential Organ Failure Assessment (SOFA) score ≥ 2 for at least one of respiratory function (ratio of partial pressure of arterial oxygen and fraction of inspired oxygen [PaO_2_/FiO_2_] < 300), liver function (bilirubin > 33 μmol/L), coagulation (platelets < 100 × 10^3^/μL) and renal function (creatinine > 171 μmol/L). Exclusion criteria included: aged < 18 years, consent could not be obtained, patient not expected to survive 24 h, known glucose-6-phosphate dehydrogenase (G6PD) deficiency, known or suspected pregnancy or breastfeeding.

The primary outcome was the patients’ vasopressor requirements expressed as mean hourly dose and duration of noradrenaline equivalents administered. Sample size calculations were derived from the means and SDs of the noradrenaline dose and duration data from the Zabet et al. RCT [5], which was the only published data available at the time. This indicated that a total of 36 participants (18 per group) would have 95% power to detect a difference in the mean norepinephrine dose over three days of 6.5 (SD 6) µg/min or a difference of 22 (SD 22) hours norepinephrine duration with 5% type 1 error. Therefore, a total of 40 participants were enrolled to account for an anticipated 10% loss due to withdrawal of consent to continue.

### Administration of intervention

A computer-generated random block size randomisation sequence was prepared in advance by the study statistician (JW). This was used to randomise the participants (1:1 ratio) to the vitamin C intervention group or placebo control group. Blinding of the treatment allocation was through sequentially numbered opaque sealed envelopes opened by the research nurses who also prepared the intervention. Finally, the intervention was dispensed to clinical staff blinded to which study arm the participants were allocated.

Patients in the active intervention group received intravenous vitamin C (ASCOR L500, McGuff Pharmaceuticals, Santa Ana, USA) in 5% dextrose at a dose of 25 mg/kg body weight every 6 h. This was made up into a 50 ml syringe and administered over 30 min via syringe pump into a peripheral or central venous line. The total dose of 100 mg/kg/day was administered for up to 96 h (duration of the study), or until death or ICU discharge if earlier. Patients in the placebo control group received intravenous 5% dextrose in water made up in identical 50 mL syringes swap to delivered and administered the same as for the intervention group.

### Collection of clinical data

Data was collected and managed using REDCap (Research Electronic Data Capture), a secure, web-based data collection and storage tool hosted at the University of Otago, New Zealand. Data was de-identified using a patient study code. The following demographic and clinical data were collected at baseline: age, gender, weight, ethnicity, primary diagnosis contributing to sepsis, comorbidities, ICU severity scores (SAPS, APACHE III, and SOFA), vasopressor dose, vital signs and oxygenation parameters. In addition, the following clinical data were collected daily: vasopressor dose delivered over time (units/min), SOFA scores (predicts ICU mortality), vital signs, oxygenation parameters, and at follow-up: length of ICU and hospital stay, and ICU and hospital mortality.

Adverse events, other than those considered part of the study inclusion process and/or study outcome assessments (e.g. organ failure, death; unless believed to be due to the intervention), were recorded as per the Common Terminology Criteria for Adverse Events (CTCAE, version 4.0).

### Collection and analysis of blood samples

Blood samples were collected daily for routine haematological and biochemical parameters (e.g. white cell counts and differentials, lactate, creatinine, C-reactive protein), and were analysed by Canterbury Health Laboratories, an International Accreditation New Zealand (IANZ) laboratory. A blood sample (4 ml LiHeparin) was also collected daily before the first vitamin C infusion. This was then processed rapidly at 4 °C for subsequent storage of acidified supernatants and plasma aliquots at − 80 °C for batch analysis of vitamin C by HPLC with electrochemical detection, as described previously [23], and for analysis of plasma myeloperoxidase using a commercial sandwich ELISA kit (AbCam, Cambridge, UK).

### Statistical analyses

The baseline characteristics were summarised descriptively and tabulated by treatment group. Plasma vitamin C concentrations were summarised by treatment group and day. The patients’ hourly vasopressor requirements post randomisation (the primary outcome of interest) were calculated as noradrenaline dose equivalent units/min (equivalent to 0.1 µg/kg/min noradrenaline) by summing noradrenaline (µg/kg/min), adrenaline (µg/kg/min), and 2.5*vasopressin (units/min) according to the formula by Goradia et al. [24]. Each patients’ total vasopressor requirements were then summarised as mean dose delivered per hour over the 96-h study period or until death if this occurred prior, and total duration of delivery. Differences in mean vasopressor dose by treatment group were assessed by unpaired t-test. Time until event data (including duration of vasopressor delivery, discharge from ICU, and discharge from hospital) was assessed using Kaplan–Meier plots and log-rank tests censoring for death. Differences in duration of vasopressor delivery over the study period (0–96 h) were assessed by comparing the restricted mean survival time (equivalent to the area under the Kaplan Meier curve) between the study groups [25]. SOFA scores were measured at 0, 24, 48 and 96 h post randomisation; patients who were discharged were given a score of 0 (the minimum) and those who died a score of 24 (the maximum). Linear models were used to estimate the change in SOFA scores over time, and the difference in change over time between groups. All statistical analyses were performed using R (4.1.1, R Core Team 2021, Vienna, Austria) [26].

## Results

### Participant characteristics at baseline

**Fig. 1:**
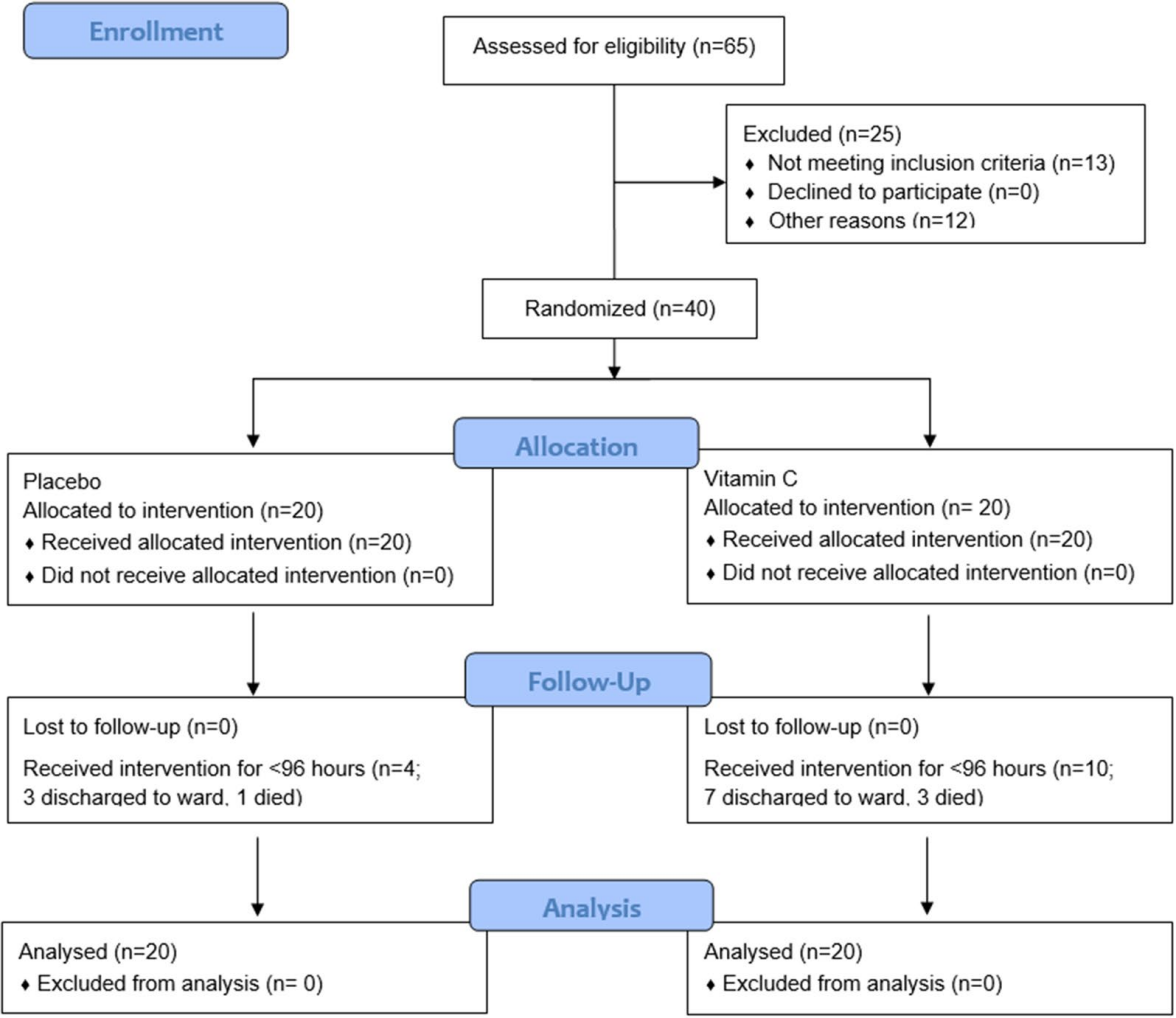
CONSORT flow diagram

Of the 65 patients assessed for eligibility, 40 were randomised to either placebo or intravenous vitamin C (Fig. 1). The participants were predominantly male (67%), aged 68 (61, 75) years and weighed 80 (72, 98) kg (Table 1). They comprised 85% European, 10% Māori/Pasifika peoples and 5% Asian/other ethnicities. The predominant source of sepsis was abdominal (35%), followed by pulmonary (23%), skin/soft tissue and blood (18% each; Table 1). The ICU severity scores (SAPS, APACHE III and SOFA) and other physiological and biochemical parameters for the whole cohort and the treatment subgroups are also shown in Table 1. The median (Q1, Q3) time from admission to randomisation was 17 (12, 25) hours and from randomisation to first treatment was 45 (33, 73) minutes.

**Table 1:** Participant characteristics at baseline

	Total (n = 40)	Intervention (n = 20)	Placebo (n = 20)
Sex (male)	27 (67)	16 (80)	11 (55)
Age (years)	68 (61, 75)	69 (64, 76)	66 (57, 71)
Weight (kg)	80 (72, 98)	80 (72, 86)	86 (70, 98)
*Source of sepsis*			
Abdominal	14 (35)	8 (40)	6 (30)
Pulmonary	9 (23)	3 (15)	6 (30)
Skin/soft tissue	7 (18)	4 (20)	3 (15)
Blood	7 (18)	3 (15)	4 (20)
Urinary tract	3 (8)	2 (10)	1 (5)
Other/unknown	3 (8)	1 (5)	2 (10)
SAPS 2	50 (41, 58)	50 (41, 59)	49 (42, 58)
APACHE-III	84 (73, 97)	85 (76, 98)	81 (70, 95)
SOFA Score	9.0 (7.0, 10)	8.5 (6.8, 11)	9.0 (7.8, 10)
Vasopressor dose (units/min) ^a^	1.4 (0.7, 2.4)	1.4 (0.7, 2.8)	1.2 (0.8, 2.3)
Core temperature (°C)	37 (37, 38)	37 (37, 38)	37 (37, 38)
Heart rate (beats/min)	98 (80, 116)	100 (89, 116)	94 (80, 113)
Mean arterial pressure (mmHg)	73 (68, 86)	73 (69, 86)	77 (76, 86)
PaO_2_	76 (65, 105)	77 (65, 109)	76 (65, 102)
FiO_2_	0.4 (0.3, 0.6)	0.4 (0.3, 0.6)	0.4 (0.3, 0.6)
PaO_2_/FiO_2_	195 (142, 309)	223 (141, 308)	188 (144, 302)
Lactate (mmol/L)	2.0 (1.2, 3.4)	2.2 (1.2, 3.1)	1.9 (1.3, 3.4)
Creatinine (µmol/L)	203 (108, 295)	222 (114, 308)	193 (97, 268)
White cell count (× 10^9^/L)	13 (7.2, 21)	8.4 (5.1, 18)	15 (12, 22)
Any comorbidity	6 (15)	3 (15)	3 (15)

Data represent n (%) or median (Q1, Q3)

*APACHE* acute physiology and chronic health evaluation, *SOFA* sequential organ failure assessment, *SAPS* simplified acute physiology score, *FiO*_*2*_ fraction of inspired oxygen, *PaO*_*2*_ arterial partial pressure of oxygen

^a^Vasopressor comprises a combination of noradrenaline, adrenaline, and vasopressin

### Vitamin C kinetics

**Fig. 2:**
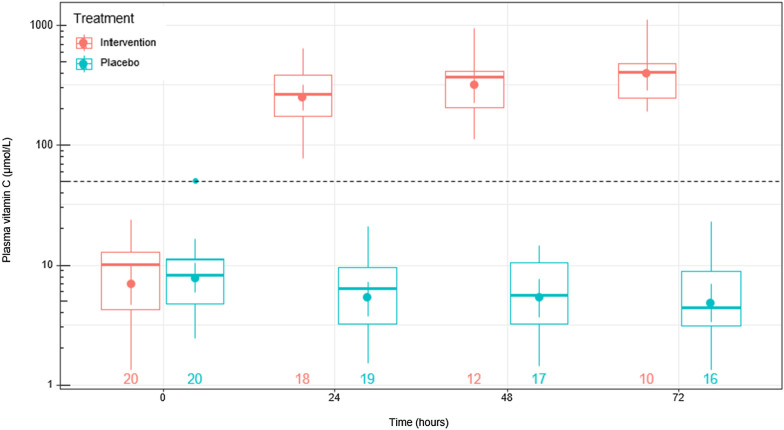
Plasma vitamin C concentrations for patients with septic shock who received intravenous vitamin C relative to placebo. Intravenous vitamin C was administered at a dose of 25 mg/kg six-hourly, and blood samples were collected daily to analyse vitamin C concentrations by HPLC. Box plots show median values with 25th and 75th percentiles as boundaries and whiskers indicate range; circles indicate mean values and 95% CI

The plasma vitamin C status of the study participants was deficient at baseline, with a median (Q1, Q3) of 9.2 (4.4, 11.4) µmol/L. Intravenous vitamin C was administered at a dose of 25 mg/kg six-hourly. The median weight of the participants was 80 kg, resulting in median administration of 2 g vitamin C six-hourly (or 8 g/day). Blood samples were collected immediately before the first intervention infusion of each day, i.e., on average, six hours post the last infusion. Within 24 h, there was a significant increase in plasma vitamin C concentrations in the intervention group from 10 (4, 13) µmol/L to 264 (159, 391) µmol/L and up to 408 (227, 560) µmol/L by 72 h (Fig. 2). In contrast, the placebo group decreased from 8.2 (4.7, 11) µmol/L to 4.4 (3.1, 8.8) µmol/L over the 72 h.

One patient with severe renal dysfunction (oliguria—urine output of 253 [197, 339] ml/day, serum creatinine of 656 (592, 806) µmol/L and estimated glomerular filtration rate of 5 [4, 7] ml/min/1.73 m^2^), had elevated plasma vitamin C concentrations (952 [643–1095] µmol/L). This was likely due to an attenuated ability of the kidneys to clear the infused doses. No adverse events were reported in association with these elevated vitamin C concentrations.

### Effect of intervention on vasopressor requirements and organ failure

**Fig. 3:**
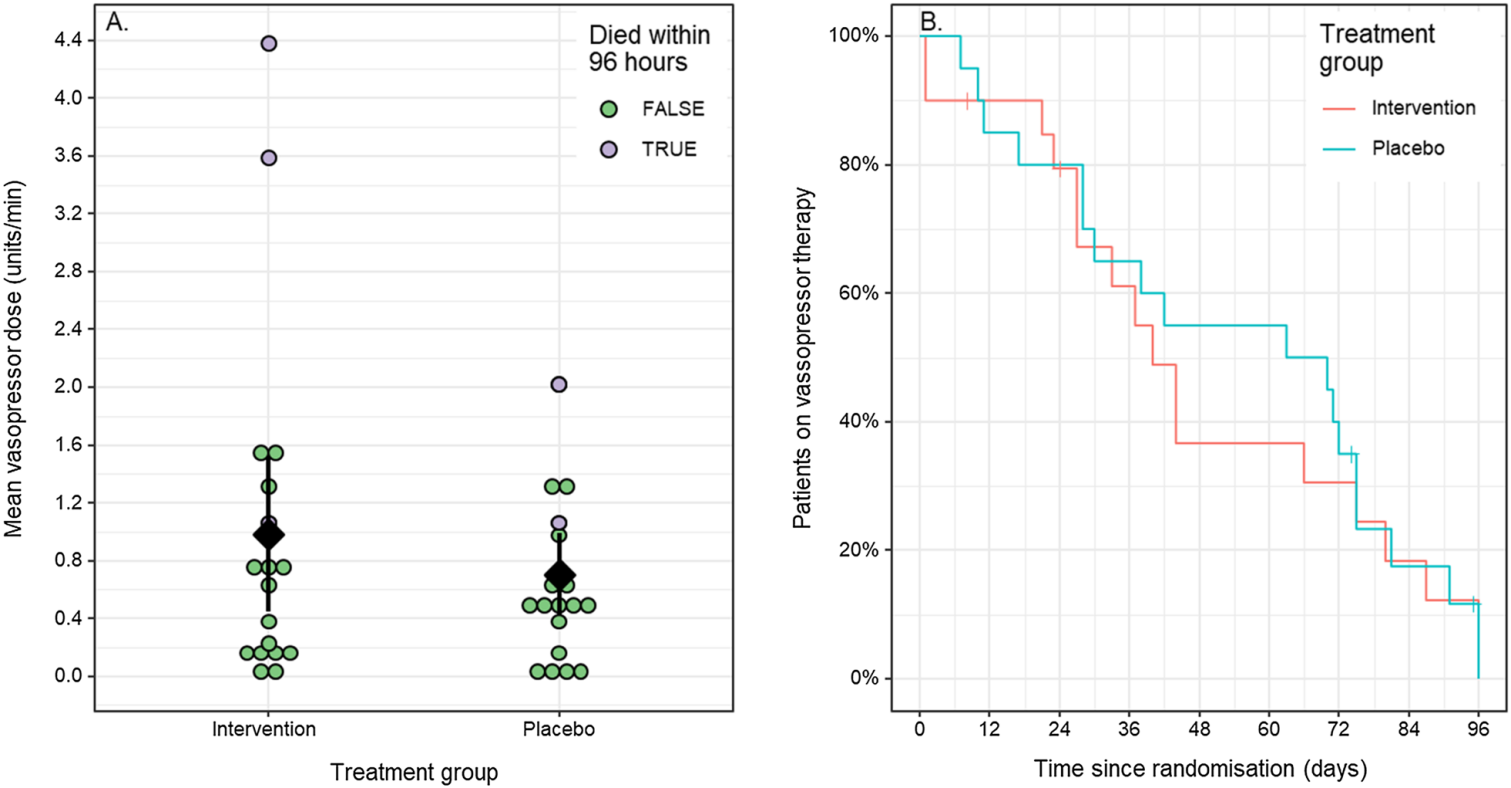
Dose and duration of intravenous vasopressors delivered to patients with septic shock by treatment group. **a** Dot plot of mean vasopressor dose (units/min) delivered over the four-day study period (96 h) or until death by treatment group. Filled circles represent mean dose for each individual patient, black diamond with vertical line represent group means with 95% CIs. **b** Kaplan Meier plot of time from randomisation until cessation of vasopressor therapy by treatment groups. There was no significant difference between the patients who received intravenous vitamin C and those who received the placebo in mean dose (p = 0.35) nor duration (p = 0.64) of vasopressor administration. See Additional file 1: Figure S1 for the individual data

Noradrenaline and other vasopressors are used to titrate blood pressure to a target mean arterial pressure of typically 65 mmHg. Vasopressor administration is decreased when the mean arterial pressure target is being met. The mean dose rate of vasopressor administered in the vitamin C group was 0.99 units/min (SD = 1.15) and in the placebo group was 0.71 units/min (SD = 0.60), a mean difference of 0.28 units/min (95% CI − 0.31 to 0.87, p = 0.35; Fig. 3a). The mean duration of vasopressor administration in the vitamin C group was 48 (95% CI 35–62) hours and in the placebo group was 54 (41, 62) hours, with a between group difference of − 6 h (95% CI − 25 to 13, p = 0.54) hours (Fig. 3b). The use of steroids could influence the dose and duration of vasopressor administration in septic shock, however, we did not observed any significant differences in the proportion of people receiving intravenous hydrocortisone between the two groups over time (p > 0.05). Mean SOFA scores decreased over time by an estimated 0.73 units per 24 h, but there was no evidence of a difference between treatment groups (p = 0.20; see Additional file 1: Figure S2). At 96 h post randomisation the mean (SD) SOFA score in the vitamin C group was 6.7 (8.3) and in the placebo group was 5.5 (7.0), an estimated difference of 1.2 (95% CI − 3.8 to 6.1, p = 0.64).

### Effect of intervention on length of stay and mortality

**Fig. 4:**
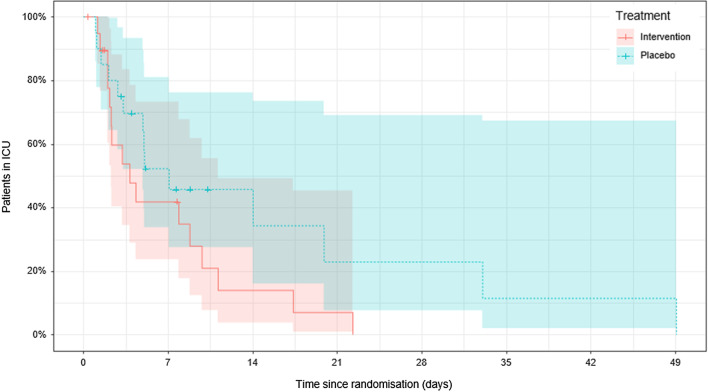
Kaplan–Meier plot for ICU length of stay of the study participants. Lines (bands) indicate the percentage of patients (95% CIs) still in ICU by time in days since study randomisation. Crosses indicate participants censored for death. There was no significant difference between the intervention and placebo groups (p = 0.12, log rank test)

The median ICU length of stay in the intervention group was 3.8 (2.2, 9.8) days versus 7.1 (3.1, 20) days in the placebo group (p = 0.12, censoring for death), a difference of − 3.3 days (Fig. 4). Median hospital length of stay in the intervention group was 18 (11, 35) days versus 22 (10, 52) days in the placebo group (p = 0.65, censoring for death), a difference of -4 days. Of the total cohort, 33% (intervention = 6, placebo = 7) of the cohort had died by 30 days and 38% (intervention = 8, placebo = 7) by 90 days; 35% (intervention = 7, placebo = 7) died in hospital. There was no difference in mortality between the two groups (p > 0.05).

### Effect of intervention on leukocytes and markers of inflammation

Leukocyte counts, particularly neutrophil counts, were elevated at baseline (13 [10, 18] × 10^9^/L). By 72 h the neutrophil counts were higher in the vitamin C group than the placebo group (20 [15, 24] × 10^9^/L vs 9 [6, 15] × 10^9^/L, p = 0.01; see Additional file 1: Figure S3). Plasma concentrations of the neutrophil enzyme myeloperoxidase were also elevated at baseline (181 [155, 247] ng/mL); the concentrations did not change significantly throughout the study, nor was there a significant difference between the two treatment groups (p > 0.05; see Additional file 1: Figure S4). Plasma concentrations of C-reactive protein, a marker of inflammation, were elevated at baseline but decreased significantly during the study, from 277 (201, 384) mg/L to 167 (94, 210) mg/L (p < 0.0001). There was no significant difference between the two treatment groups (p > 0.05; see Additional file 1: Figure S5).

### Adverse events

Only one adverse event (gastrointestinal bleed) was reported; this was not related to the intervention as the participant was in the placebo group.

## Discussion

We carried out a pilot RCT to ascertain if intravenous vitamin C administration to patients with septic shock would decrease their vasopressor requirements relative to placebo. Overall, we did not observe a significant difference in dose or duration of total vasopressor (noradrenaline, adrenaline and vasopressin) administration between the two groups over the duration of our study. This is in contrast to the Zabet et al. RCT [5], which had reported a significant decrease in noradrenaline dose and a 22-h decrease in the duration of noradrenaline administration (mean of 50 vs 72 h; p = 0.007) in their cohort of septic patients treated with a comparable dose of intravenous vitamin C (100 mg/kg/day). Two other trials administered intravenous vitamin C at a dose of 6 g/day and also reported a significant decrease in duration of vasopressors (55 vs 156 h, p = 0.001, and 26 vs 44 h, p < 0.05) [8, 9]. Another trial administered 60 mg/kg/day vitamin C as a continuous infusion to critically ill patients with pneumonia and reported a decrease in vasopressor use from 82 to 55 h (p = 0.003) [10]. Further RCTs that have used vitamin C in combination with hydrocortisone and/or thiamine have also reported improvements in vasopressor requirements [27–29]. It is interesting to note that all seven trials were carried out in low-middle income countries (Iran, Egypt and India). A review of global vitamin C status and prevalence of deficiency indicated that low-middle income populations tend to have lower vitamin C status and a higher prevalence of deficiency than high-income populations [30]. Thus, vitamin C intervention may be more effective in countries whose populations are likely to be already chronically insufficient in vitamin C prior to the acute deficiency induced by severe sepsis.

In our study, we observed a non-significant trend towards a shorter ICU length of stay of − 3.3 days in the vitamin C group (3.8 vs 7.1 days, p = 0.12) and a non-significant decrease in hospital length of stay of − 4 days (18 vs 22 days, p = 0.65). Recent meta-analyses have indicated that vitamin C administration to critically ill patients may decrease ICU length of stay [31, 32]. The median cost per ICU stay in Australia and New Zealand is > $4,000 per day, with an average length of stay of 10 days for septic patients (a total of > $40,000 per ICU stay) [33, 34]. Thus, a decrease in ICU length of stay of several days would positively impact the economic burden of septic shock on the healthcare system.

Administration of intravenous vitamin C at a dose of 25 mg/kg q 6 hourly to the septic patients (equating to 2 g q 6 hourly) resulted in a significant increase in median plasma vitamin C concentrations from deficient values (9.2 µmol/L) to 264 µmol/L within 24 h and up to 408 µmol/L by 72 h. Other studies have reported comparable plasma vitamin C concentrations for doses of 1.5 g q 6 hourly, 12.5–50 mg/kg q 6 hourly, and 1–5 g q 12 hourly [4, 21, 35]. Elevated vitamin C concentrations (952 [643–1095] µmol/L) were observed in one patient with severe renal dysfunction due to an attenuated ability to clear the intravenous doses. However, no adverse events related to the vitamin C infusion were reported. Nevertheless, elevated vitamin C concentrations can interfere with some point-of-care-glucose monitors resulting in spurious values and potential for overly aggressive insulin administration in some cases [36].

Markers of inflammation (e.g. C-reactive protein, myeloperoxidase) were elevated in the patients at baseline. However, there were no significant differences between the two groups over time. We did observe a significant increase in neutrophil counts in the vitamin C group relative to placebo by 72 h (20 vs 9 × 10^9^/L, p = 0.01). Neutrophils are a primary innate immune defence against invading pathogens and are typically elevated during sepsis (reference range: 1.9–7.5 × 10^9^/L). Although our observation may be an artefact of fewer patients in the vitamin C group relative to the placebo group at this time point (n = 9 vs n = 16), there are also mechanistic rationales for this finding. Despite the higher neutrophil counts in the vitamin C group, there were no significant differences in plasma concentrations of the neutrophil enzyme myeloperoxidase between the two groups. This may indicate decreased necrotic cell death of the neutrophils in the vitamin C arm. We, and others, have previously shown that vitamin C decreases NETosis, a form of neutrophil necrotic cell death [37–39]. Cell-free DNA, a marker of necrotic cell death, has been detected in patients with sepsis and is decreased in those treated with intravenous vitamin C [40, 41]. An early RCT in septic surgical patients has also indicated decreased neutrophil apoptotic cell death in patients treated with low dose intravenous vitamin C [42]. Thus, vitamin C may be decreasing necrotic and apoptotic cell death and thereby increasing the lifespan of neutrophils in septic patients.

There are several limitations of this study. First, the size of the study was likely too small to detect the clinical outcomes. The sample size for the primary outcome of vasopressor requirements (dose and duration) was determined from the Iranian Zabet et al. trial [5]. However, a subsequent meta-analysis of the effects of vitamin C on atrial fibrillation has indicated that trials carried out in Iran gave overly positive results compared with trials carried out in the US [43]. This may be due to the populations of low-middle income countries tending to have lower vitamin C status and a higher prevalence of deficiency than those from high-income countries [30]. Thus, higher participant numbers may be required to detect differences in cohorts from high-income countries who are not chronically insufficient in vitamin C. Another limitation may be the dose of vitamin C administered. At the time of ethics submission (2016), only two small vitamin C and sepsis RCTs had been published, the Fowler et al. trial (n = 24), which had administered 50 and 200 mg/kg/day vitamin C [4], and the Zabet et al. trial (n = 28) which had administered 100 mg/kg/day vitamin C [5]. Because fewer patients had been treated with the higher dose of vitamin C, the ethics committee asked that we halve our vitamin C dose to 100 mg/kg/day due to safety concerns, despite evidence in other diseases states indicating tenfold higher doses being safe [44]. Subsequent studies using vitamin C doses of 200 mg/kg/day and up to 25 g/day have shown potential effects on mortality in critically ill patients and an excellent safety profile [11, 12]. Of note, higher vitamin C doses may be required in septic patients due to potential downregulation of cellular vitamin C transporters in response to inflammatory mediators, thereby potentially attenuating cellular uptake of infused vitamin C [45]. A further limitation was the length of time between ICU admission and initiation of intravenous vitamin C administration (median of ~ 18 h). Sepsis is a very time-sensitive condition, with earlier intervention being associated with better patient outcomes [46]. A retrospective study of time sensitive mortality difference with vitamin C combination therapy indicated a significant decrease in mortality in participants who received the intervention within six hours of sepsis presentation relative to those who received it after more than six hours [47]. Furthermore, the duration of vitamin C administration is an important consideration for long-term clinical outcomes [48], with intervention for five or more days showing greater benefit [49]. Half of the intervention group in our trial did not receive the entire four days of intravenous vitamin C. Previous research has indicated that cessation of vitamin C infusions after 2–4 days can result in some patients reverting to hypovitaminosis C concentrations [11, 21]. Thus, overall, it appears that our dosing regimen may have been ‘too little, too late’ to see clinical benefits.

## Conclusions

In contrast to the Zabet et al. study [5], our pilot study did not show statistically significant decreases in dose or duration of vasopressor infusion. However, larger studies that take into account the intervention timing, dose and duration limitations described above, as well as the potential impact of country of trial, may provide more definitive evidence.

## Supplementary Information

**Additional file 1**. Supplemental material: Figure S1–S5.

## Data Availability

The datasets generated during the current study are available from the corresponding author upon reasonable request for meta-analyses.

## Abbreviations

APACHE: Acute Physiology and Chronic Health Evaluation
ICU: Intensive care unit
SAPS: Simplified Acute Physiology Score
SOFA: Sequential Organ Failure Assessment
