# Supplementary material for: Intravenous vitamin C administration to patients with septic shock: a pilot randomised controlled trial
Source: Crit Care. 2022 Jan 25;26:26. doi: 10.1186/s13054-022-03900-w (PMC8786621; doi:10.1186/s13054-022-03900-w)
Supplement: Supplementary file 1 — Additional file 1. Supplemental material: Figure S1–S5. [file 13054_2022_3900_MOESM1_ESM.pdf]

Supplemental data

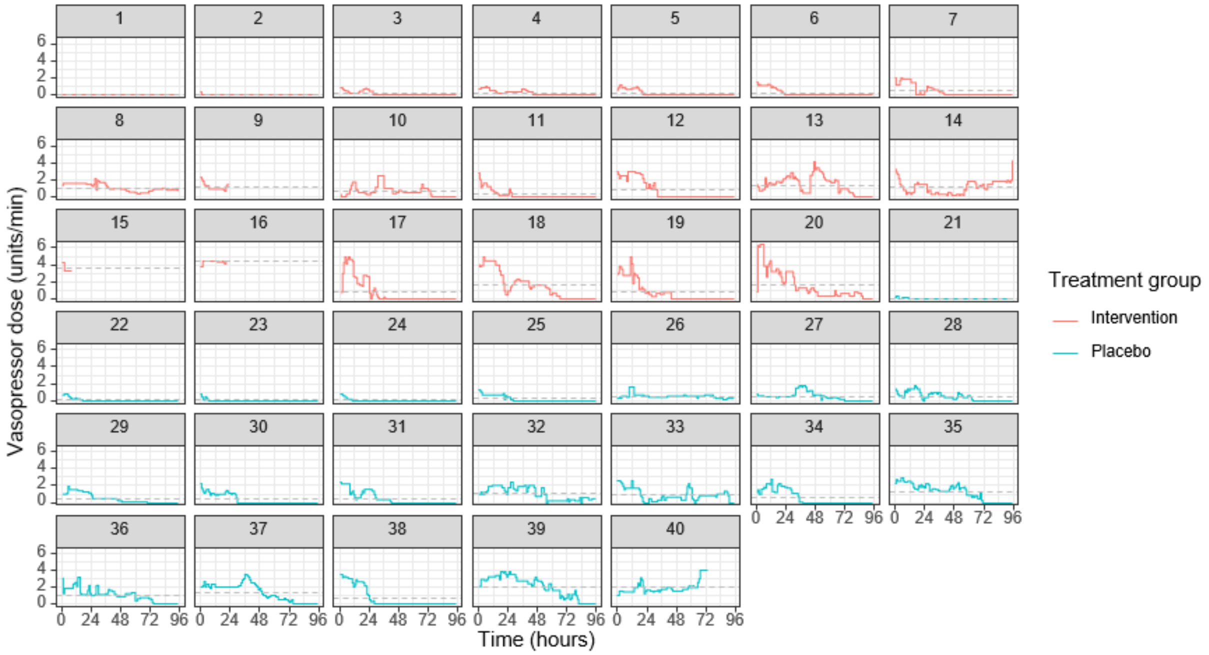

Figure S1. Individual changes in vasopressor treatment over time. Vasopressor comprised noradrenaline, adrenaline and vasopressin.

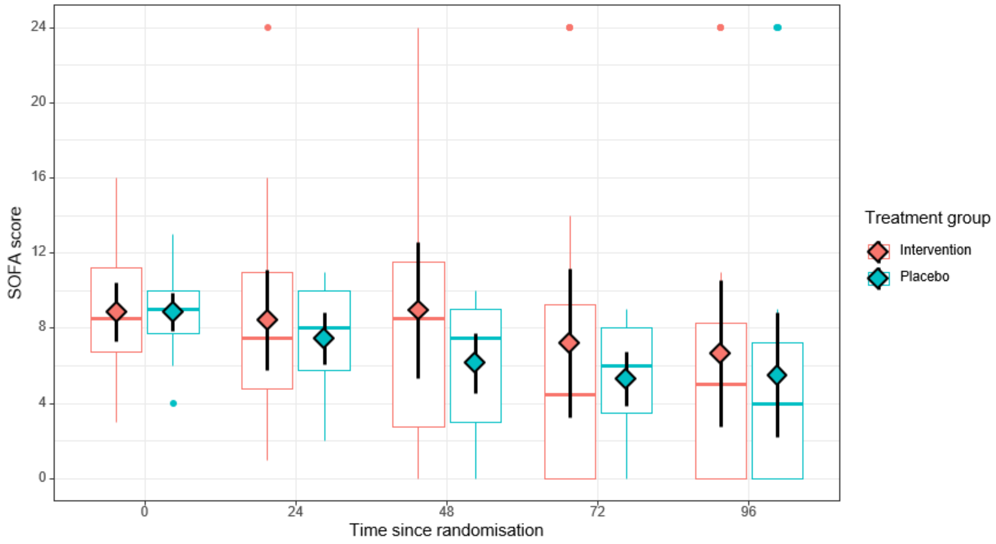

Figure S2. Effect of vitamin C intervention on SOFA scores of the septic patients. Box plots show median values with 25th and 75th percentiles as boundaries and whiskers indicate range; diamonds with vertical lines represent group means with 95% confidence intervals. There was no significant difference between the two groups ($p > 0.05$).

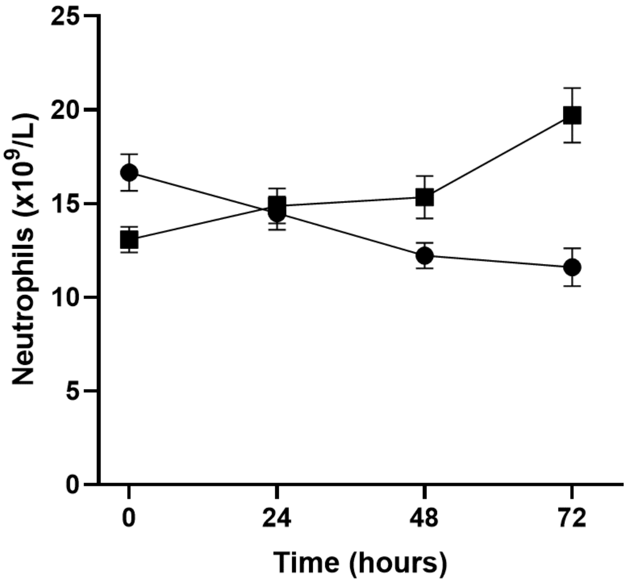

Figure S3. Effect of vitamin C intervention on neutrophil counts of the septic patients. Vitamin C intervention (100 mg/kg/day) is represented by the squares (■) and placebo infusion is represented by the circles (●). Data represent mean and SEM. Mixed effects analysis indicated a trend towards increasing neutrophil counts in the intervention group and decreasing neutrophil counts in the placebo group ($p = 0.07$ for both). There was a significant difference between the two groups at T = 72 hours ($p = 0.01$).

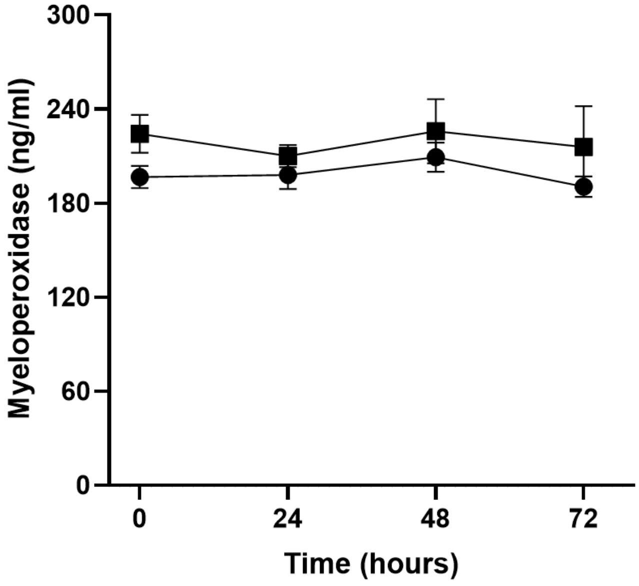

Figure S4. Effect of vitamin C intervention on plasma myeloperoxidase concentrations of the septic patients. Vitamin C intervention (100 mg/kg/day) is represented by the squares (■) and placebo infusion is represented by the circles (●). Data represent mean and SEM. There was no significant difference in myeloperoxidase concentrations over time between the two groups ($p > 0.05$).

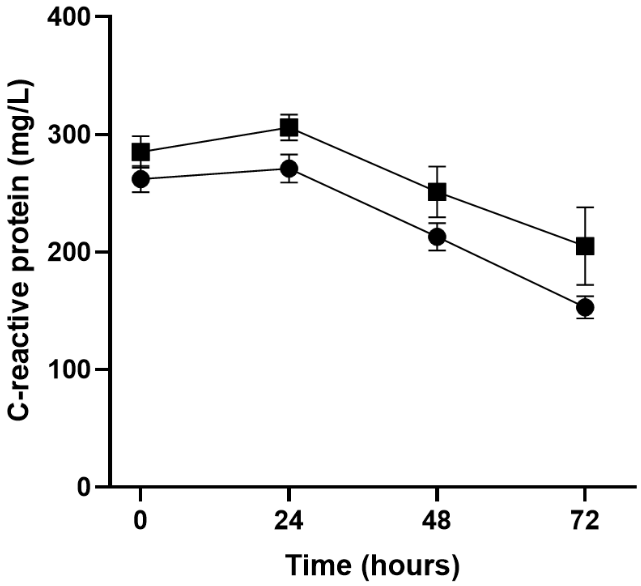

Figure S5. Effect of vitamin C intervention on plasma C-reactive protein concentrations of the septic patients. Vitamin C intervention (100 mg/kg/day) is represented by the squares (■) and placebo infusion is represented by the circles (●). Data represent mean and SEM. There was no significant difference in change of C-reactive protein concentrations between the two groups ($p > 0.05$).
